# Supplementary material for: Framework for Participatory Quantitative Health Impact Assessment in Low- and Middle-Income Countries
Source: Int J Environ Res Public Health. 2020 Oct 21;17(20):7688. doi: 10.3390/ijerph17207688 (PMC7589915; doi:10.3390/ijerph17207688)

**Policy Brief**

**September 2020**

**
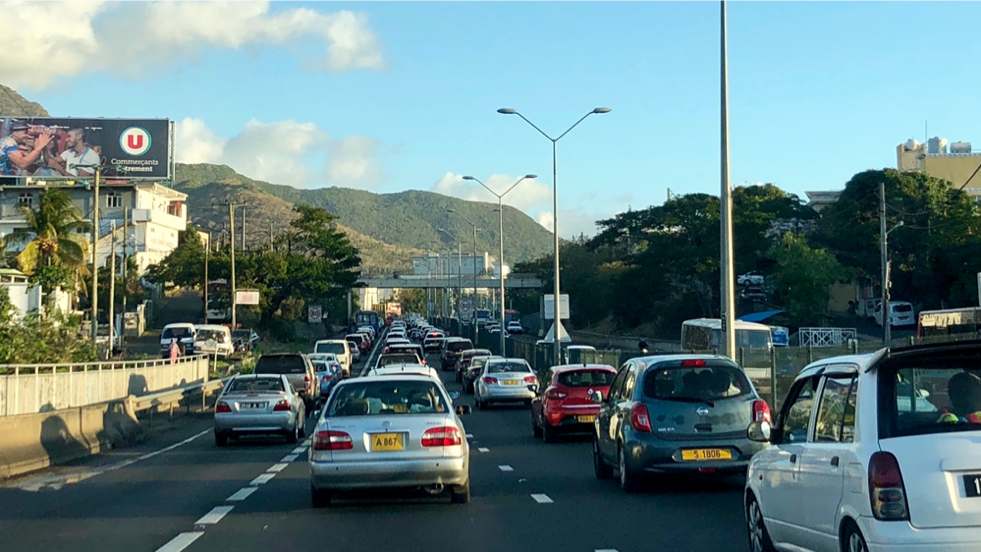
**

**A new approach to Urban Transport**

In a rapidly urbanising world, cities must consider the health impacts of urban policies and projects. This policy brief makes a strong case for participatory quantitative Health Impact Assessments (HIAs), a promising and practical tool to ensure healthy, equity-driven and sustainable cities. In Mauritius, urban population rates have doubled in the last fifty years and vehicle ownership has increased by 625 % in only twenty years. There are five towns across the island, but 60% of the population lives on 8% available land. The concentration and multiplication of transport networks and settlements in restricted urban corridors causes overcrowded housing, increased congestion and cumulative poor transport planning.

**Transport and Public Health in Port Louis**

Port Louis faces major traffic congestion caused by over 200,000 people commuting to the city on working days. Most of them use private motorisation on the motorway that cuts through the city and do not use different integrated transport modes (for eg. no bus or cycle lanes) on its axis. Similar to other African coastal cities, Port Louis transport planning fails to respond to population and spatial growth with heterogenous vehicles using limited and non-adapted road infrastructure. Despite the introduction of a recent metro rail system, Port Louis remains a car-oriented city with very little green space. Pedestrian movement is also limited to unsafe sidewalks, and traffic flow is hampered by narrow roads and side-street parking spaces.

So, what happens if motorisation further increases? Health risks in cities like Port Louis are likely to grow as well – and the health status of the adult population in Mauritius is already characterised by a strong prevalence of diabetes, hypertension and cardiovascular diseases. There is a risk for physical inactivity to increase, as people choose motorised modes instead of walking and cycling. Also, levels of air pollution and traffic deaths are likely to rise.

**PQHIA in Port Louis**

Participatory Quantitative HIAs (PQHIA) use statistical data and risk assessment modelling to estimate health risks while engaging stakeholders. The aim was to understand and estimate how proposed transport policies would impact the health of residents.

**Steps of PQHIA in Port Louis**

●**●** Several stakeholders across the public and private sector were engaged in order to design and conduct a local model of PQHIA.

●**●** Participants were interviewed about local urban transport policies and provided their views on what health meant to them, and on the potential health impacts of urban transport.

●**●** Information from the stakeholders were combined with policy reviews, the data on travel patterns, air pollution, traffic deaths and physical activity.

●**●** Health impacts of different transport mode shifts in Port Louis were estimated using a risk assessment approach that compares a reference scenario (the current situation) with three alternative scenarios.

●**●** All scenarios, types of policies and the associated challenges were built in collaboration with stakeholders during focus groups discussions.

**Results**

Policies to reduce car use on a large scale will not be enough to save lives. One alternative scenario included a strong reduction in both car and motorcycle use indicates health benefits through the increase in physical activity, decrease exposure to air pollution and reduce traffic fatalities. In terms of economic impact, this hypothetical scenario resulted in reductions worth 23 million US Dollars due to the number of averted traffic deaths. Car-dependent designs strongly contradict the main mean of mobility adopted by more than half of urban population in Port Louis: walking. Strong, rather than mild policies targeted at different modes of motorisation – and not only the reduction of private cars will be useful to orient transport agendas towards health and social benefits. It is important to maintain and increase the protective effects of active travel modes such as walking and cycling in Port Louis.

**Policy and advocacy recommendations**

**▶▶** Urban transport must be tackled as an opportunity to encourage physical activity

**▶▶** Transport policies should aim to restrict all forms of private motorized vehicles and promote active and public transport to support public health

**▶▶** Policies must include specific restrictions for motorcycle traffic.

**▶▶** Policies promoting the benefits of physical activity should be accompanied by interventions to increase pedestrian and cyclists’ safety

**▶▶** Policies to increase public transport use should provide incentive for users of private motorized modes.

**Summary**

Using a participatory approach to conduct HIAs in settings such as Mauritius is valuable, and should be conducted in other cities, too. Participatory quantitative HIAs can improve the development of healthy and sustainable transport networks while encouraging stakeholders to actively contribute to the future of their cities. HIAs can facilitate dialogue, bring local knowledge to the table, and use scientific data modelling methods to generate powerful impact estimations. Participatory approaches can help stakeholders to understand the HIA process and encourage them to use HIA outcomes in the field of urban development, climate change and, ultimately, in favour of people and planet.

**For more information please contact:** Meelan Thondoo - [mthondoo@isglobal.org](mailto:mthondoo@isglobal.org).


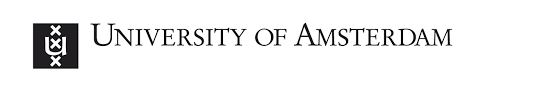

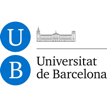

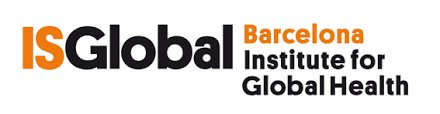

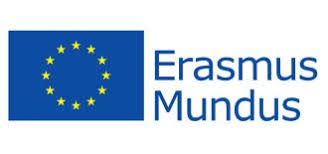

Supplement: Supplementary file 1 [file ijerph-17-07688-s001.zip › Supplementary_Files/Annex B_PolicyBrief_2609.docx]
