# Supplementary material for: Framework for Participatory Quantitative Health Impact Assessment in Low- and Middle-Income Countries
Source: Int J Environ Res Public Health. 2020 Oct 21;17(20):7688. doi: 10.3390/ijerph17207688 (PMC7589915; doi:10.3390/ijerph17207688)
Supplement: Supplementary file 1 [file ijerph-17-07688-s001.zip › Supplementary_Files/Annex A_Fieldwork Protocol_2009.docx]

**Fieldwork Protocol**

Participatory Quantitative HIA

2017-2020

Table of Contents

[1. Mortality Pathways for Urban and Transport Policies 2](#_Toc51460979)

[2. Fieldwork Model 3](#_Toc51460980)

[3. Category of Stakeholders for Step 1/ Screening and Step 2/Scoping 4](#_Toc51460981)

[4. List of communities of Interest 5](#_Toc51460982)

[5. Tentative Activity Log 5](#_Toc51460983)

[6. Stakeholder Analysis Table (Worksheet) 6](#_Toc51460984)

[7. Data Sourcing 6](#_Toc51460985)

[8. Quantitative Data by Categories 7](#_Toc51460986)

[9. Budget 9](#_Toc51460987)

[10. Individual Interviews Semi-Structured Topic Guide 10](#_Toc51460988)

[11. Focus Group Discussion Semi-Structured Topic Guide 12](#_Toc51460989)

[12. Validation Exercise Semi-structure Topic Guide 13](#_Toc51460990)

#
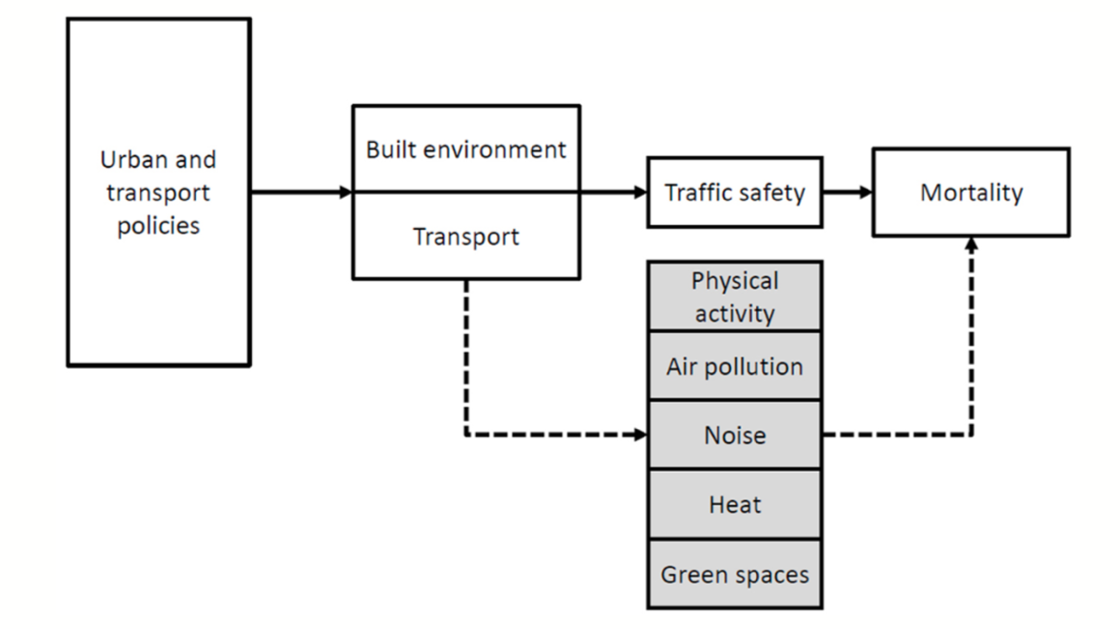
Mortality Pathways for Urban and Transport Policies

**Figure 1.** Conceptual framework of the Urban and Transport Planning Health Impact Assessment (UTOPHIA) tool. (*1*) Recommended exposure level; (*2*) current exposure level; (*3*) exposure difference between recommended and current exposure level; (*4*) exposure response function (ERF) quantifying association between exposure and mortality; (*5*) relative risk (RR) corresponding to exposure difference; (*6*) population attributable fraction (PAF) corresponding to exposure difference (Mueller, 2017).

# Fieldwork Model


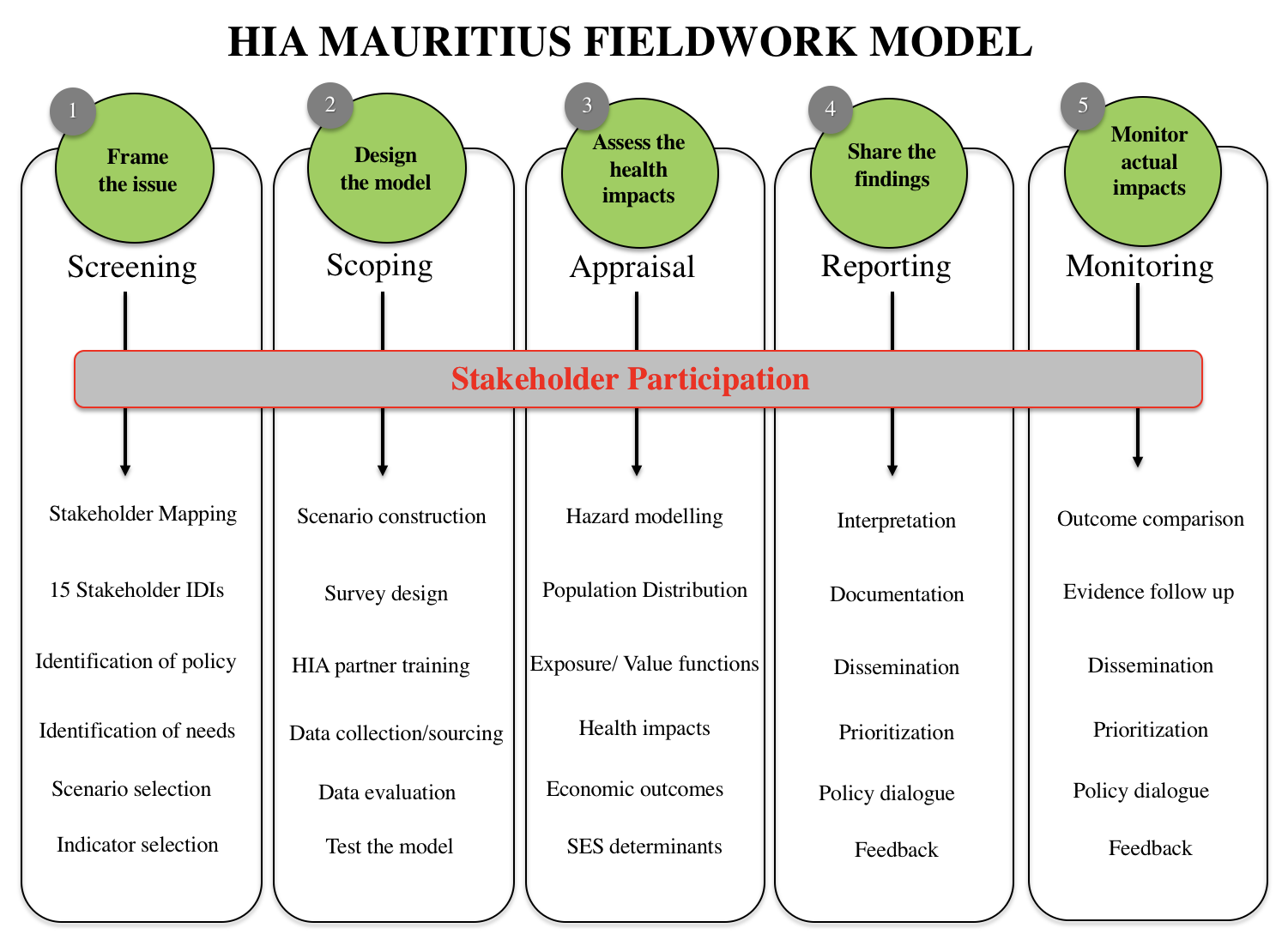


Phase 1: The purpose of the first phase (issue framing) is to identify and enter into dialogue with adequate political and social partners. Stakeholder consultations will be held in order to define barriers and opportunities for HIA in respective study settings. The importance of consulting stakeholders is to (1) define the current status of HIA in the study setting (2) identify the ongoing or prospective urban and transport planning policies and (3) to build realistic and context-specific scenarios overtime.

Phase 2: The aim of design stage is to deliver a specific protocol for conducting the impact evaluation. This part will focus on the execution of integrated full-chain HIAs of urban and transport planning policies in Port Louis and Manhiça City. A novel HIA model and framework will be constructed in order to estimate the health impacts of scenarios of selected urban and transport planning policies. This implies clearly defining the system and protocol that best fits for analytical procedures: exposing clear variables and relationships and presenting solid reference and alternative scenarios.

Phase 3: The execution stage will involve conducting the evaluation assessment by using modelling techniques. The HIA will include the traditional risk assessment steps of hazard identification, exposure assessment and risk characterization. It will assess the combined effect of environmental and social factors on health with newly collected or existing national data (see the data collection section below). The models ITHIM and UTHOPIA that are proposed have been tested, used, and validated in other studies (Woodcock, 2011; Muller 2015). Sensitivity analysis will be conducted to assess uncertainties.

Phase 4: The appraisal stage is the result dissemination phase. The HIA process and outcomes from Mauritius will be compared to those of Mozambique, with the aim of discussing commonalities and differences in health determinants and impacts. This part will analyse how HIA can be integrated in local policy processes and contribute to sustainable development and achievement of SDG 2030.

Phase 5: The monitoring phase consists of establishing HIA procedures and assessing the feasibility of evaluating and monitoring estimated health impacts. This phase is illustrated here but may be out of the scope of the project given the 3-year time limit.

# Category of Stakeholders for Step 1/ Screening and Step 2/Scoping

| Category of Stakeholder | Opportunities | Challenges |
| --- | --- | --- |
| Elected Officials | - Provide information on political objectives, timelines and decision-making processes - Inform on current opportunities and feasibility to apply recommendations - Provide guidance on how to address concerns of policy-makers | - Hard to access and limited time availability - Need to educate on HIA process - Politically constrained - May not support the use of HIA outcomes |
| Experts from Public Agencies | - Provide data and analysis on health, land use, housing and socio-economic situation - Prepare forecasting reports and monitoring impacts - Bridge to policy-makers and potential leaders in HIA practice | - May not be interested in health - Lack of technical and financial capacity - May not want to participate on a long-term basis - May be concerned about HIA outcomes |
| Residents | - Grassroots data and results - Potential to mobilize community leadership - Help to address language, knowledge and cultural barriers | - Irregular or insufficient engagement due to time constraints and varying interest - Lack of trust in researchers, agencies or projects - Capacity building measures required - Potential need to provide incentives |

# List of communities of Interest

| Community based organization |
| --- |
| Residents |
| Elected officials at municipal, regional, state/provincial level |
| Small businesses |
| Industry, developers, and big business, Service providers |
| Public agencies |
| Statewide or national advocacy organisations |
| Academic, learning and research institutions |
| HIA consultant organizations |

# Tentative Activity Log

| Date | Time | Where | Affiliation | Purpose |
| --- | --- | --- | --- | --- |
| Tues  07Aug | 11:30-12:30 | Bureau of Statistics | N/A | Access Road Transport and Road accident statistics |
| Tues  07Aug | 14:00-15:30 | MRC | Academia1 | Intro HIA |
| Wed  08Aug | 14:00-15:00 | Bureau of Statistics | GovtOff1 | IDI1 |
| Wed 22 Aug | 10:30- | Traffic planner, CNT, Vacoas, Bonne Terre | ServiceProv1 | IDI2 |
| Wed  22Aug | 21:00- | Restaurant BG | Exp1UP | IDI5 |
| Thurs  23Aug | 12:00- | Restaurant PL | Industry1 | IDI8 |
| Thurs  23Aug | 16:30- | PH | CBO2 | IDI6 |
| Thurs  23Aug | 20:00- | Restaurant FF | Citizen1 | IDI7 |
| Lun 27Aug | 11:00- | PH | Industry2 | IDI12 |
| Tue  28Aug | 9:00- | S.WilliamNewton st, Moorgate House, 9^th^ floor | GovtOff2 | IDI9 |
| Tue 28 Aug | 13:00- | EDB 10^th^ floor | PubAgency1 | IDI3 |
| Fri 24 Aug | She returns | TBD | Exp1Engineer | IDI10 |
| Fri 24 Aug | 16:00- | PH | CBO1 | IDI4 |
| Thurs 23 Aug | He returns | TBD | AdvOrg1 | IDI11 |
| Mon 27 Aug | 10:00 | Ministry Building | GovtOff2 | IDI13 |
| Tuesday 28 Aug | 11:00 | TBD | CBO3 | IDI14 |

# Stakeholder Analysis Table (Worksheet)

| Stakeholder group | Representative (contact info) | Expertise (information held) | Role in HIA | Interest or concerns about HIA* | Power to influence policy/development* | Opportunities to communicate (when,where) |
| --- | --- | --- | --- | --- | --- | --- |
| Elected Officials |  |  |  |  |  |  |
| Public Agency |  |  |  |  |  |  |
| Residents |  |  |  |  |  |  |

Table adapted from Baker et al. (2011)

Using these two a prioritization assessment can be created in categories: with those A) interested in HIA and with influence, B) non-interested in HIA with influence, c) interested no influence, D) non-interested, no influence. With this an extra effort will be done to contact those in categories A, B, and secondary C.

# Data Sourcing

| **Type** | **Purpose** | **Source** | **Indicators/ Target** |
| --- | --- | --- | --- |
| **Qualitative data** | Build scenarios | **15 IDIs** with stakeholders | 1. Perceptions of health in city 2. Vision for healthy and sustainable U&T policies 3. Potential of HIA to impact on policy making |
|  |  | **1 FGD** (narrative evaluation) |  |
| **Quantitative data** | Collect baseline exposure data | **380 Surveys**  **Local databases**  National statistics  Census  Hospital records  City Council records  Police records  Spatial maps of land use  Climate monitoring station  **International databases**  WHO Air Pollution database  Climate monitoring station  DHS  Global Burden of Disease  NDVI | 1. Demographics (Age, Sex, SE status) 2. Burden of disease 3. Causes of deaths 4. Levels of Physical activity 5. Travel Patterns 6. Distance to public transport 7. Road traffic deaths 8. PM 2.5 9. Green space 10. Noise 11. Heat |

# Quantitative Data by Categories

|  | **Categories** | **Inputs to modelling** | **Examples of data sources** | **Suggestions of places to look for** |
| --- | --- | --- | --- | --- |
| 1 | Demographics | Population by gender and age | Census | National institute of statistics |
|  |  |  | Intercensal survey |  |
|  |  |  | Intercensal estimate |  |
| 2 | Travel patterns | Mode and time of travel by gender and age,  Length travelled, distance travelled. | Household travel survey (sometimes called mobility survey) | Transport agencies, consultancies, academic research |
|  |  |  | Census | National institute of statistics |
|  |  |  | Travel demand model | Transport agencies, consultancies, academic research |
|  |  |  | Physical activity survey | Health or sport agencies, academic research |
| 3 | Air pollution | Concentration of PM2.5, fraction due to road transport, emission by mode of transportation, and concentration of PM2.5 in the subway | On-road measurement of PM2.5 pollution | WHO Global Urban Ambient Air Pollution Database, environmental agencies, academic research |
|  |  |  | EDGAR (modelled) estimates of PM2.5 | EU Emissions Database for Global Atmospheric Research |
|  |  |  | Source apportionment reports | WHO Database on Source Apportionment Studies for Particulate Matter in the Air, environmental agencies, academic research |
|  |  |  | Emission inventory of road transport | Environmental agencies, academic research |
| 4 | Physical activity | Energy expenditure on non-travel physical activity, by gender and age  MET hr/week | Health survey | WHO STEPS, health or sport agencies, academic research |
|  |  |  | Sports and recreation survey |  |
|  |  |  | Physical activity survey |  |
|  |  |  | Movement sensors |  |
| 5 | Road injuries | ‘Who-hit-whom matrix’ for deaths and injuries, by gender and age | Traffic collisions records | Traffic police, transport agencies |
|  |  |  | Vital registration statistics | National institute of statistics, health agencies |
|  |  |  | Mortuary and burial registers | Local mortuaries |
|  |  |  | Household health and injury survey | Health and transport agencies, academic research |
|  |  |  | Hospital records | Local hospitals, health agencies |
| 6 | Burden of disease | Deaths, years of life lost (YLL) and years lost due to disability (YLD) by cause, gender and age | Vital registration statistics | National institute of statistics, health agencies, Health statistics outcomes |
|  |  |  | Burden of disease data | WHO Global Health Observatory data, Global Burden of Diseases, Injuries, and Risk Factors Study, other academic research |
| 7 | Heat | Daily mean temperature- 1 year (average mean/day in C·)  99th versus 74th temperature percentile | Climate monitoring station | Mauritius Meteorological station  World Meteorological Organization |
| 8 | Green space | Map of land use (industrial lots, residences, green space)  Per 10% increase in greenness  Street network, topography layers, public transport layers, households layer, census track | NDVI | University research  Parastatal agencies: Landscope, EDB |
| 9 | PM 2.5 | Per 10 μg/m3 increase in PM2.5 exposure | Air quality monitoring stations | WHO Air Pollution database  National Environmental Laboratory of the Department of Environment |
| 10 | Noise | Daytime traffic noise LAeq,16hr | Monitoring stations |  |

# Budget

| **Survey Data Collection** |  |  |  |  |  |
| --- | --- | --- | --- | --- | --- |
|  | **Item** | **Number of items** | **Price per item** | **Total MUR** | **Total EUR** |
|  | Fieldworker salaries | 8 | 12000 rps/fieldworker | 96000 | 2412 |
|  | Bus travel for FW | 20 days | 400 rps/day | 8000 | 201 |
|  | Wkend bonus | 8 | 1000 rps/wkend | 8000 | 201 |
|  | Software & hosting | One-off fee | 24000 rps | 24000 | 603 |
| **Intern Support** |  |  |  |  |  |
|  | Intern support Part 1 YR | 20hrs | 20hrs | 1875 | 47* |
|  | Intern support | 20hrs | 20hrs | 1500 | 38* |
| **FGD** |  |  |  |  |  |
|  | Room location | 2hrs | 750 | 1500 | 38* |
|  | Facilitation strategy | 3hrs | 1000 | 3000 | 75* |
|  |  |  |  |  |  |
| **Data Costs** |  |  |  |  |  |
|  | Heat data | 5 | 200rps | 1000 | 25 |
|  | Cartography Layers | 4 | 4000 | 16000 | 402 |
|  | Transport | 40hrs | 100rps/hr | 4000 | 101* |
|  | Outline Planning Scheme | 1 |  | 5000 | 126 |
|  |  |  |  |  |  |
|  |  |  |  | Total | 4268 |

# Individual Interviews Semi-Structured Topic Guide

**Objective of the IDIs:**

1. What is current status in urban and transport policies?
2. What is important to them?
3. Do they think that UTP (urban & transport planning) is related to health -if yes, how?
4. What is their idea of a healthy and sustainable UTP system?
5. What is needed to achieve that?
6. What is feasible?
7. What is missing in the current situation? (is there overuse of motor-vehicles, over-isolation from car-use, no consciousness about pollution, like or dislike)
8. What would make your personal behavior change to more healthy or sustainable actions?

**Recall Aim of Stakeholder consultation**

🡪 Consultation= important process for decision makers to anticipate the consequences of their decisions

🡪 Inclusion of diverse stakeholders enhances HIA core values: democracy, equity, sustainable development & ethical use of evidence

Identify important stakeholder concerns

Assemble experiences, knowledge, expertise

Create support for implementation of HIA recommendations

Shape HIA communication & dissemination methods

**Recall the objective of the HIA:**

🡪 Assess an existing policy to (1) estimate its impact on health and (2) assess whether improving it can promote sustainable development.

*Note: Maybe sustainability has positive health outcomes. Maybe health is a co-benefit of sustainability*

| **BACKGROUND**   - Describe the role of your group/institution/employer concerning urban & transport planning - Are you active, involved in projects, measure – what are your responsibilities? - With whom do you cooperate (Traffic, Transport, Mobility sector, Health sector) ? |
| --- |

| **TOPIC 1: HEALTH MEANING**   - What do you think makes you healthy in the city? (more specific) - How do you manage your health while in the city? (health and transport more specifically) - Do you think that UTP is related to health -if yes, how? - Do you think there are needs in terms of health to support transport/urban decision making? - If yes, how can health can be used to support urban/transport decision making? |
| --- |

| **TOPIC 2: UTP POLICIES**   - **Can you describe 2 major U&T policies you are familiar with? (current legislation)** - Involvement of interviewee - Involvement of group/institution/employer - **How do you think these policies may impact (+/-) on health in the city?** - Policies- promoting health? - Do you think health was considered when shaping such policies? - Which importance had the arguments related to “health”? - How were they implemented? - **About the policy itself** - Name of the measure - Which measure? - Where? When? (time frame: short, medium, long term) - What is/was the aim? Which results are/were expected? - Did the results happen? Has the measure been evaluated? - Who (person or institution) had the idea to implement this measure? Who was involved (persons, city, district, public participation)? Who was mainly responsible for the project? Responsibilities? - How did they finance the project? What lessons have been learnt? Have there been any supporting factors or barriers? - Do you have any data available about this measure (e.g. counts of cyclists, pedestrians, accident data, etc.)? Could you provide any documents? |
| --- |

| **TOPIC 3: HIA**   - Do you know about Health Impact Assessments? - Yes: Have you ever used it (or other experiences)? - No: Can you imagine using it? For which purpose? - To what extent do you think HIA outcomes will be taken seriously in decision-making? - How do you think HIA can support sustainable development? |
| --- |

| **TOPIC 4: VISION & WISHES**   1. What is your idea of a healthy and sustainable UTP system in PL? 2. What is needed to achieve that? (which measures to be implemented to promote) 3. What is feasible? What framework conditions would that require? 4. Why have good ideas and measures failed so far? 5. What is missing in the current situation? (is there overuse of motor-vehicles, over-isolation from car-use, no consciousness about pollution, like or dislike) 6. What would make your personal behavior change to more healthy or sustainable actions? 7. How could sectors/groups/departments cooperate better? 8. How should it happen?   Note: inform about current exposure levels and shift towards indicators of interest |
| --- |

# Focus Group Discussion Semi-Structured Topic Guide

Duration: 1.5hrs maximum

Number of people: 3-5 maximum at a time

Prerequisite for engagement: IDI completed

**Main objective of the Panels:**

contrast perspectives and opinions between stakeholders

engage small-scale dynamics between experts, public officials, and citizens

co-create the research agenda: finalize the three scenarios together

**Stakeholders are invited to:**

share their individual stories and express their needs and priorities (10%).

share their opinions about the 3 proposed scenarios of a healthy and sustainable UTP system (10%)

discuss if, where and how their individual visions differ and clash with the 3 scenarios (60%)

discuss if they can reach similar endpoints (10%)

**Potential Structure of the Panels:**

Introduction of each stakeholder to one another

Overview of the 3 scenarios emerging from IDIs

Constrast-facilitation session

# Validation Exercise Semi-structure Topic Guide

| Duration | Action | Concern |
| --- | --- | --- |
| 3 mins | Short welcome and updates | What has been done since our last exchange? |
| 4 mins | Reporting of baseline exposure data and final HIA results | What data has been used and what are the health impact assessment outcomes? |
| 4 mins | Relevance of HIA outcomes to their positions and fields | What is the relevance of the findings to you?  What were the expectations on the HIA outcomes?  How would you prefer to see the HIA outcomes?  How can/ will you use the HIA outcomes/outputs? |
| 4 mins | Re-integration of HIA results in the society | What are the conditions necessary to integrate HIA results in your own sector/agency?  What are the barriers and opportunities to integrate HIA results in your own sector/agency?  Who should receive this information?  When should this information be shared to have greater impact? |
| 5 mins | Feedback on participatory HIA process | What do you think of the process and engagement in the HIA?  Were your expectations met?  How can we increase your attention/interest on future HIA?  What do you need from HIA experts to support their future work? |
| 5 mins | Open floor for questions and comments |  |
